# Lifestyle Recommendations in the Dutch Clinical Practice Guidelines

**DOI:** 10.1002/gin2.70037

**Published:** 2025-09-04

**Authors:** Florien Ham, Anh N. Nguyen, Elizabeth F. C. van Rossum, Mariëtte R. Boon, Janke F. de Groot

**Affiliations:** ^1^ Knowledge Institute of the Federation of Medical Specialists Utrecht the Netherlands; ^2^ Department of Internal Medicine, Division of Endocrinology Erasmus MC, University Medical Center Rotterdam Rotterdam the Netherlands

**Keywords:** chronic disease management, clinical practice guidelines, lifestyle

## Abstract

**Introduction:**

Medical specialists play an essential role in guiding lifestyle changes in chronically ill patients, contributing to health‐related prevention. Clinical practice guidelines (CPGs) are an important tool to support medical specialists to implement health‐related prevention in daily practice. However, it remains unclear how recommendations on lifestyle interventions are part of CPGs. Therefore, the objective of this study was to analyse existing lifestyle recommendations in Dutch CPGs for medical specialists.

**Methods:**

Using a mixed‐methods approach, the study examined 130 CPGs to assess the prevalence, type of lifestyle factors being addressed, level of evidence according to GRADE, knowledge gaps and wording of lifestyle recommendations.

**Results:**

Results indicated that 65% of the examined CPGs included lifestyle recommendations, with increasing physical activity and improving nutrition being the most addressed factors. Despite only 11% of the recommendations being supported by moderate to high certainty of evidence, 62% were strongly formulated. At the same time, the analysis also revealed unclarity in the wording of recommendations regarding who, what and when to act. Finally, this study shows notable knowledge gaps regarding the effectiveness and feasibility of lifestyle interventions in daily practice.

**Discussion:**

Despite robust evidence, lifestyle recommendations do have a prominent position in CPGs for chronic diseases in specialist' care. Further research is needed to answer to the knowledge gaps and to what is needed for lifestyle recommendations to be implemented in daily practice.

## Summary

- Lifestyle recommendations were included in 65% of chronic disease CPGs, highlighting their clinical relevance.
- 62% of the lifestyle recommendations were strongly worded, but often lacked clarity on who should act, what to do, and when—limiting practical utility.
- There is a notable knowledge gap regarding the real‐world effectiveness and feasibility of lifestyle interventions in daily clinical practice.
- The findings underscore the need to understand whether, why, and how lifestyle advice is implemented in routine care.
- Guideline development should focus on clearer phrasing and stronger links to implementation evidence.

## 1 Introduction

Over 33% of the global population suffers from chronic diseases such as cardiovascular diseases, cancer or diabetes [1]. The Dutch healthcare registration identified diseases with a high disease burden, mortality rate and healthcare costs. These diseases are cancer, cardiovascular diseases, respiratory diseases, depression, obesity, skin diseases, rheumatic diseases, diabetes mellitus and dementia, which are a large burden on the healthcare system [2]. Hereby, accessibility, affordability and sustainability are challenged.

Almost all chronic diseases are associated with unhealthy lifestyle behaviours, such as poor diet, smoking and lack of physical activity [3]. In 2050, the rising global burden of lifestyle‐related (chronic) diseases will impact healthcare systems and society significantly, with increased healthcare costs, higher prevalence of chronic conditions, and exacerbation of health inequities [4]. While people are still expected to live longer, the age of onset of chronic disease will decrease, therefore increasing the number of years living with care needs [5]. To prepare healthcare systems for current and future challenges concerning chronic disease management, it is important to note that many chronic diseases are at least in part preventable [6, 7].

Prevention can be categorized into two distinct levels, each targeting different populations and involving different stakeholders. The first level concerns collective prevention and refers to the protection and promotion of health of the general population (universal prevention) or specific groups within the general population at risk for poor lifestyle and early detection of diseases (selective prevention) [8]. The second level concerns individual prevention and refers to either the prevention of disease worsening or recurrence in people with a disease (health‐related prevention) or the prevention of developing lifestyle‐related disease in people with an increased risk for certain diseases (indication‐related prevention) [9]. Recommendations for individual prevention are described in national clinical practice guidelines (CPGs). Given their formal status, recommendations on lifestyle in CPGs are an effective tool to implement health‐related and indicated related prevention in medical care [10]. In the context of individual health‐related and indicated related prevention, lifestyle interventions are defined as a sustainable lifestyle change throughout life [11].

Medical specialists play a pivotal role in guiding lifestyle changes in chronically ill patients, who are often more open to such advice during recurring hospital visits—creating repeated ʻteachable momentsʼ [12]. Unlike primary care physicians, who have an established role in health‐related prevention, Dutch medical specialists have only recently begun to engage more actively in this area, as outlined in the 2035 vision document [13]. Still, their attitudes, knowledge, and roles in addressing lifestyle factors vary widely [14]. To clarify this responsibility, we propose that CPGs can offer essential guidance by outlining best practices based on current evidence. Dutch CPGs are developed by 32 associations of medical disciplines within the Dutch Federation of Medical Specialists and are created by multidisciplinary working groups [15].

The aim of this article was to perform a cross‐sectional analysis of medical specialist' CPGs for chronic diseases to reveal the prevalence, content, evidence and wording of lifestyle recommendations. With this aim, we applied a mixed‐methods methodology combining descriptive quantitative strategies and inductive/deductive qualitative strategies.

## 2 Methods

### 2.1 Research Design

This mixed‐methods study combined descriptive quantitative and inductive/deductive qualitative approaches to gain a comprehensive understanding of lifestyle recommendations in CPGs for medical specialists. We employed a multi‐layered analytical framework, illustrated in Table 1.

**Table 1:** Multi‐layered analytical framework.

Variable	Retrieved from	Approach	Analytical framework
Prevalence: Lifestyle recommendations	Recommendations from published CPGs concerning predefined disease groups.	Descriptive quantitative strategy	Lifestyle was defined as a sustainable lifestyle change throughout life, addressing the following lifestyle factors: physical activity, smoking, alcohol/substance use, nutrition, relaxation, sleep, psychosocial care and generic lifestyle advice [11]. Predefined disease groups: Cancer, cardiovascular diseases, respiratory diseases, depression, obesity, skin diseases, rheumatic diseases, diabetes mellitus and dementia [18].
Content: Lifestyle factors	Lifestyle recommendations	Exploratory sequential design	Deductive coding strategies categorized recommendations into the lifestyle factors being addressed: physical activity, smoking, alcohol/substance use, nutrition, relaxation, sleep, psychosocial care and generic lifestyle advice.
Evidence: Level of evidence	Lifestyle recommendations	Descriptive quantitative strategy	According to the GRADE handbook, five levels were distinguished: high, moderate, low, very low and no GRADE [16].
Evidence: Knowledge gaps about lifestyle.	Knowledge gaps from the retrieved CPGs including lifestyle recommendations.	Explanatory and exploratory sequential design	Inductive coding strategies were applied to gain insight into the content of the knowledge gaps concerning lifestyle.
Wording: Strength	Lifestyle recommendations	Exploratory sequential design	Deductive coding strategies categorized recommendation into strong, weak or no recommendations, based on GRADE's assumptions for wording of recommendations [17].
Wording: Additional elements	Lifestyle recommendations	Exploratory sequential design	Inductive coding strategies were applied to further distinguish essential elements for wording of recommendations.

### 2.2 Context of Research

Dutch CPGs for medical specialists are developed and maintained by 32 associations of medical disciplines, the majority in collaboration with the Dutch Knowledge Institute of the Federation of Medical Specialists [15]. Dutch CPGs follow the methodology of the Grading of Recommendations Assessment, Development and Evaluation (GRADE) working group to assess the certainty of the body of evidence [16] and considerations from clinical practice by the so‐called ‘evidence‐to‐decision framework’ [17]. The general structure of CPGs is illustrated in the supporting materials (Figure S1). Dutch CPGs for medical specialists are published online through a CPG database (https://richtlijnendatabase.nl/) and describe the best care based on the best available evidence from literature and practice. Dutch CPGs are developed by multidisciplinary working groups, involving medical specialists, general practitioners, patient organizations and other stakeholders. A guideline methodologist supports the process by offering methodological, structural and procedural guidance.

### 2.3 Data Sources and Selection Process

In this cross‐sectional analysis, we systematically reviewed all the recommendations and considerations concerning lifestyle interventions in the Dutch CPGs for medical specialists in a predefined set of CPGs. This set was based on existing rankings on the occurrence of the disease group, years of (healthy) life lost and disease burden, according to the National Institute for Public Health and Environment [18]. The following disease groups were selected: cancer, cardiovascular diseases, respiratory diseases, depression, obesity, skin diseases, rheumatic diseases, type 1 and type 2 diabetes mellitus and dementia.

### 2.4 Data Extraction and Analysis

On February 1, 2024, published CPGs of the selected disease groups were exported to a data extraction form in Excel 2016, which was prepared by two authors (A.N. and F.H.). Within each CPG, the search bar of the CPG database was used to search for lifestyle recommendations by using the established list of lifestyle factors and synonyms (Table S1). All CPGs including at least one recommendation about one or more of the predefined lifestyle factors were selected for analysis. Because CPG chapters could consist of ≥ 1 recommendation, analyses were performed on the level of the individual recommendations.

### 2.5 Data Analysis

We applied different formulas to calculate prevalence, content and evidence of lifestyle recommendations. These formulas can be found in the supporting material (Figure S2). Calculations were performed by using descriptive statistics (in Microsoft Excel version 16.92, Microsoft Corporation, 2021).

Before calculating content and evidence of lifestyle recommendations, we established different themes by deductive and inductive coding strategies, according to Clarke and Braun [19]. Deductive coding was applied to reveal the lifestyle factors (content) and strength (wording) of recommendations. The knowledge gaps (evidence) and additional linguistic elements (wording) of recommendations were identified by inductive open coding strategies.

We analysed all lifestyle recommendations, applying an exploratory sequential design that quantified emerging themes. Saturation was assessed retrospectively, with no new codes or themes arising in later stages, indicating thematic completeness. To ensure coding alignment, two researchers (F.H. and A.N.) jointly coded a subset in collaboration with an experienced qualitative researcher (J.d.G.) to create an initial coding framework. The remaining data were independently coded, with consensus meetings held to resolve ambiguities and refine the framework, enhancing consistency and shared interpretation.

- Content of lifestyle recommendations
  Content of lifestyle recommendations refers to the lifestyle factors being addressed in the recommendations. Lifestyle was defined as a sustainable lifestyle change throughout life, addressing the following lifestyle factors: physical activity, smoking, alcohol/substance use, nutrition, relaxation, sleep, psychosocial care and generic lifestyle advice [11].
- Evidence of lifestyle recommendations
  Evidence of lifestyle recommendations were represented by the certainty in the body of evidence and the knowledge gaps about lifestyle.
  For assessing the certainty in the body of evidence, recommendations were categorized into the following levels, according to the GRADE handbook: no GRADE, very low GRADE, low GRADE, moderate GRADE or high GRADE [16]. CPGs from before 2012 were based on the Evidence‐Based Guideline development as proposed by the AGREE 2 framework [20] using other categories to assess the certainty in the body of evidence [21]. To summarize the level of evidence, we translated every level to a GRADE level (shown in Table S2).
  The knowledge gaps about lifestyle were identified by reading the knowledge gaps section from each chapter including at least one lifestyle recommendation (F.H. and A.N.). Knowledge gaps were included for thematic analysis when the topic concerned at least one of the predefined lifestyle factors.
- Wording of lifestyle recommendations.
  Wording of recommendations was represented by the strength of recommendations and by the additional linguistic elements. Strength was categorized, according to the GRADE handbook, into strong recommendations, weak recommendations and no recommendations. Additional linguistic elements were obtained by open coding strategies, as described earlier.
- Sensitivity analyses
  To examine relationships within our findings, we performed two sensitivity analyses. First, we examined whether the GRADE levels of evidence have improved over time, assuming increased availability of supporting literature over time. Second, we examined the relationship between the GRADE level of evidence and the strength of recommendations, as stronger recommendations are expected to align with higher evidence certainty. For both analyses, Spearman's rank correlation coefficients (ρ) were calculated using R (version 4.4.3; R Foundation for Statistical Computing). To estimate 95% confidence intervals (CIs) for the correlation coefficients, non‐parametric bootstrap resampling with 1000 replicates was performed. Confidence intervals were obtained using the bias‐corrected and accelerated (BCa) and percentile methods. Effect sizes were interpreted using Cohen's (1998) guidelines: < 0.10 (negligible), 0.10–0.29 (small), 0.30–0.49 (moderate) and ≥ 0.50 (large). Statistical significance was set at *p* < 0.05.

## 3 Results

**Figure 1:**
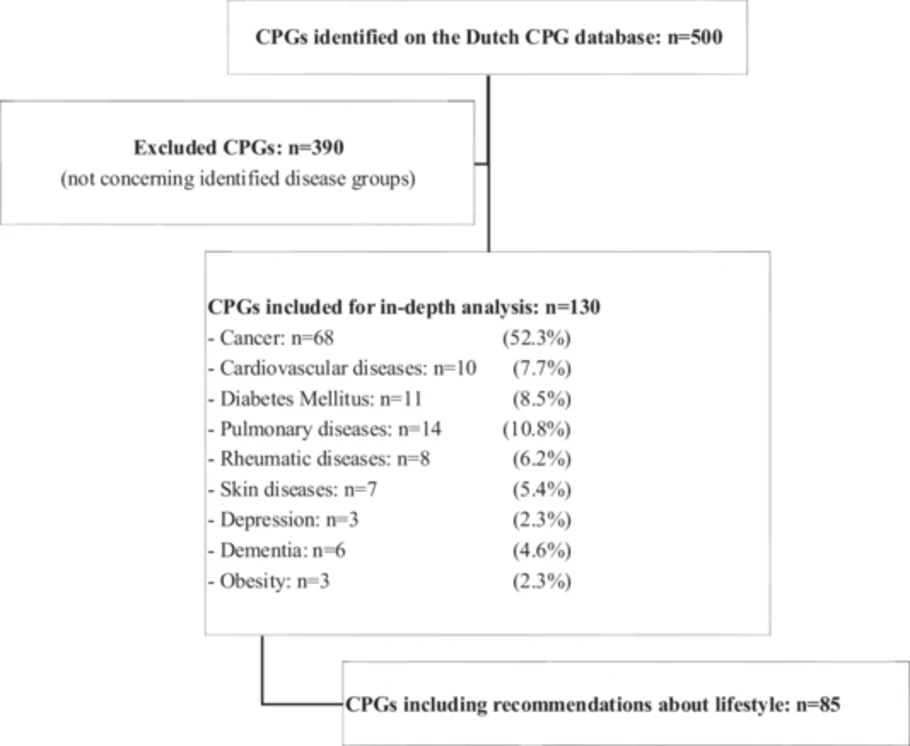
Overview of the CPGs selected for the in‐depth analysis. CPGs, clinical practice guidelines.

On February 1, 2024, the CPG database included 500 CPGs in total. 130/500 CPGs were selected for the in‐depth analysis, according to the preselected disease groups. An overview of the included CPGs and the distribution of the disease groups is shown in Figure 1. From the 130 CPGs included, 85 CPGs (65%) included lifestyle recommendations. In total, 422 individual lifestyle recommendations were extracted from the 85 CPGs.

- Content of lifestyle recommendations
  Lifestyle recommendations mostly addressed the lifestyle factors physical activity and nutrition (resp. 35% and 34%). Advice about alcohol/substances use, relaxation and sleep promotion were the least common (respectively 3%, 3% and 2%). Figure 2 shows the distribution of the lifestyle factors across all recommendations extracted from the 85 CPGs (*n* = 422). Generic lifestyle advice mostly concerned promoting a healthy lifestyle in general or a healthy weight.
  **Figure 2:**
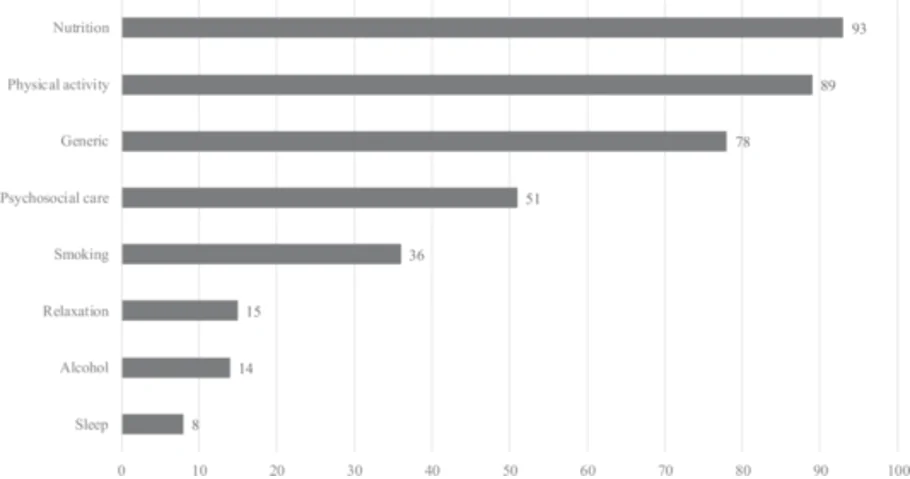
Lifestyle factors mentioned in all recommendations including lifestyle advice (*n* = 422).
- Evidence of lifestyle recommendations
  Most of the identified recommendations were not supported by evidence (66% *no GRADE*, 13% *very low GRADE* and 10% *low GRADE*). A small minority (11%) of the identified recommendations were supported by either *high GRADE (2%) or moderate GRADE (9%)* conclusions. *No GRADE* conclusions included both recommendations where no systematic literature search had taken place (50%) or by methodological downgrading (16%).
  From the chapters including lifestyle recommendations, we retrieved 33 knowledge gaps from 12 CPGs. After applying thematic analysis using an open coding strategy to these knowledge gaps, we distinguished the following knowledge gaps:
  1. Efficacy of lifestyle interventions (24/33), divided by
    a. Training interventions (*n* = 5/24)
    b. Dietary interventions (*n* = 5/24)
    c. General or combined lifestyle interventions (*n* = 11/24)
    d. Psychosocial interventions (*n* = 3/24)
  2. Predictive value of disease‐/patient characteristics (3/33)
  3. Correlation between lifestyle and disease‐related outcomes (2/33)
  4. Feasibility of dietary interventions (2/33)
  5. Effect parameters for assessing the value of physiotherapy (1/33)
  6. Frequency of monitoring vitamin status (1/33).
- Wording of lifestyle recommendations
  From our inductive and deductive coding strategies, we identified the four main themes which we then used to build a framework to further categorize and interpret individual recommendations. The next paragraph includes a description of the categories per theme, sometimes illustrated by the four example recommendations presented in the textboxes below.
  1. Strength
    This theme distinguishes three categories reflecting their strength (*strong*, *weak* and *no recommendation*). Most recommendations were coded as *strong* (*n* = 266; 62%), characterized by a directive formulation that begins with an action verb or conveys a clearly stated course of action from the working group (e.g. ***Recommendation 1 and 4***). A smaller proportion of the recommendations were *weak* (*n* = 109; 26%), characterized by being more descriptive and non‐directive in tone, including words like ʻconsiderʼ (e.g. ***Recommendation 2***). The remaining recommendations (*n* = 53; 12%) were characterized as *no recommendation*. These entries include general conclusions or reflections that do not translate into any actionable guidance (e.g. ***Recommendation 3***). Figure 3 shows the distribution and coding criteria for each category.
    **Figure 3:**
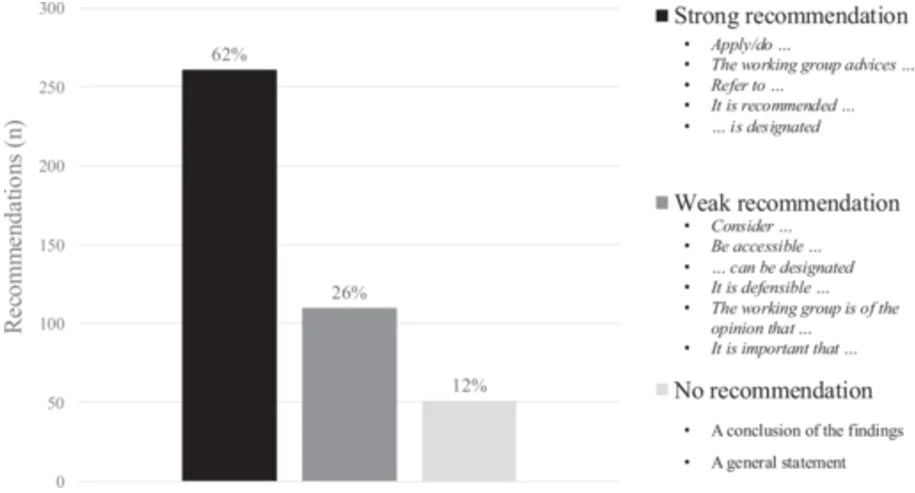
Overview of the strength of the recommendations.
  2. Action
    This theme distinguishes nine action categories. The most common type involved *carrying out an intervention* (*n* = 126; 30%), such as medical treatments or diagnostics. Other common actions concerned *advising* (*n* = 63; 15%) and *referring* to another healthcare professional (*n* = 54; 13%). Notably, 54 (13%) lacked specific action, but contained more descriptive or concluding remarks (e.g. ***Recommendation 4***). Figure 4 shows the percentage distribution and coding details for each action type.
    **Figure 4:**
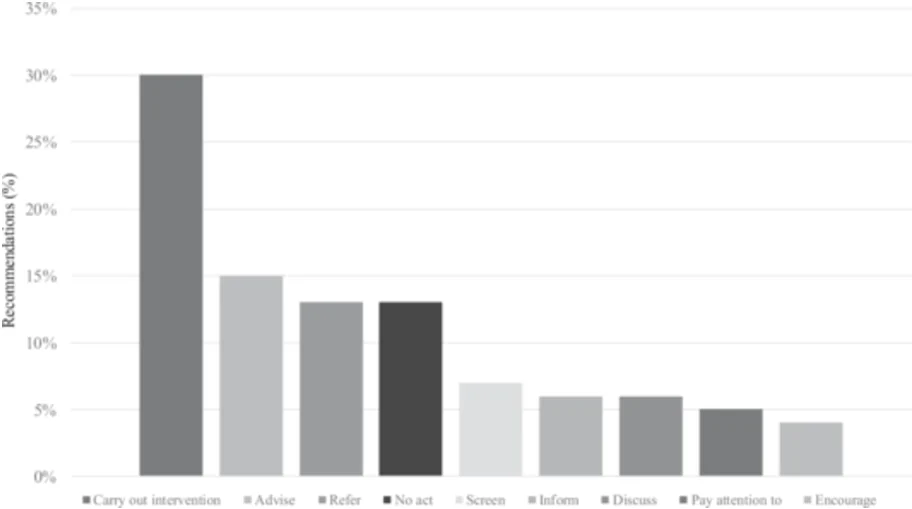
Overview of the action of the recommendation (listed from high to low prevalence): Carry out intervention (treat, offer, deploy, describe, apply, accompany, support), Advice (advise, discourage, recommend); Refer (involve, refer, guidance by, enable, collaborate); No act (strive for, be responsible for, make sure, be aware of, have knowledge about); Screen (monitor, signal, screen, control, take a test, observe, perform assessment, prosecute, be alert on); Inform (discuss, ask for, determine together, involve parents); Discuss (learn, psycho‐educate, provide information, inform, provide education, point out); Pay attention to (pay attention to, have an eye for, approach, manage, follow‐up); Encourage (encourage, stimulate, promote, motivate give exercise, strive for, council).
  3. Actor
    This theme distinguishes five actors: *medical specialist, another healthcare professional, the patient, a combination of healthcare professional and patient* and *unclear*. As each guideline targets medical specialists, recommendations were classified accordingly when the context indicated this actor. For example, in ***Recommendation 1***, the responsibility for providing an explanation is clearly directed at the medical specialist.
  4. Indication
    This theme distinguishes three indications: *the target group of the CPG, a subpopulation within the target group of the CPG* and *unclear*. Most recommendations were linked to the general target group based on the CPG's focus, even if not explicitly stated. Subpopulation recommendations referred to specific conditions or risk groups (e.g. ***Recommendations 2–4***) or relied on clinical judgement (e.g. ***Recommendation 1***).

***Recommendation 1***

**Table:** 

**Strong recommendation**	***Explain*** [*ACTION 1*] that a ***physiotherapist*** [*ACTOR 1*] or ***exercise therapist*** [*ACTOR 2*] can provide support with exercise and, ***if necessary,*** [*INDICATION*] ***refer*** [*ACTION 2*] the patient to a suitable physiotherapist or exercise therapist.
	*Source:* Dutch Orthopaedic Association, 2021 [22]

***Recommendation 2***

**Table:** 

**Weak recommendation**	***Patients who are at high risk of insufficient exercise and who appreciate supervision*** [INDICATION] can be ***referred*** [ACTION] to an appropriate exercise intervention, ***with or without specialist medical*** [ACTOR] rehabilitation.
	*Source:* Dutch Association of Rehabilitation Physicians, 2018 [23]

***Recommendation 3***

**Table:** 

**No recommendation**	To prevent further increasing the ***risk of gastrointestinal malignancies*** [INDICATION], it is important not to smoke, limit alcohol consumption, maintain a healthy diet and exercise sufficiently.
	Source: Dutch Association for Radiotherapy and Oncology, 2016 [24]

***Recommendation 4***

**Table:** 

**Strong recommendation**	The working group recommends that ***all professionals involved*** [ACTOR] ***encourage*** [ACTION] ***cancer patients*** [INDICATION] to remain active during treatment, within the limits of what is possible.
	Source: Dutch Association of Rehabilitation Physicians, 2018 [25]

- Sensitivity analyses
  The first sensitivity analysis revealed a statistically significant but negligible to small positive correlation between publication date and the GRADE level of evidence (ρ = 0.18, 95% CI [0.08–0.28], *p* < 0.001, *N* = 422), indicating a slight trend towards higher‐quality evidence in more recent publications, though with limited practical relevance.
  The second sensitivity analysis revealed a nonsignificant and negligible to small positive correlation between the GRADE level of evidence and the strength of the recommendations (*ρ* = 0.044, 95% CI [–0.06, 0.14], *p* = 0.368), suggesting that recommendation strength was largely independent of the level of evidence in this data set.

## 4 Discussion

Because lifestyle interventions are known to be beneficial for (secondary) burden, progress and quality of life in patients with chronic diseases [26, 27, 28], the purpose of this study was to analyse the recommendations on lifestyle in Dutch medical specialists' guidelines. First, we studied whether guidelines included recommendations on this topic. In addition, we analysed the content, evidence and wording of these recommendations.

This cross‐sectional analysis revealed that lifestyle recommendations were present in 65% of the preselected Dutch CPGs for prevalent (chronic) diseases. This percentage is significantly higher than a previous cross‐sectional study showing 25% of NICE CPGs including lifestyle recommendations [29]. However, we cannot directly compare the findings between the two studies because of key differences in the scope and methods. First, the guidelines selected for inclusion and in‐depth analysis varied across the two studies: In our inventory, only guidelines concerning prevalent (chronic) diseases were included, while Albarqouni [29] included all NICE Pathways available. Second, the definition of a lifestyle recommendation differed across the two studies. In the analysis of Albarqouni [29], the following lifestyle factors were included: physical activity or exercise, diet, tobacco use and alcohol use. In our analysis, lifestyle also included psychosocial care as part of lifestyle change, relaxation and general lifestyle interventions. The inclusion of a broader definition of lifestyle was a conscious choice, as mental well‐being, stress management and social care are crucial determinants of (disease‐related) quality of life that are not addressed by medical treatment [30, 31]. Even so, further analysis showed recommendations about physical activity (35%) and diet (34%) were most prominent, while recommendations about alcohol consumption, relaxation and sleep hygiene were less common (respectively in 3%, 3% and 2% of the CPGs).

In addition, this article added an extensive qualitative analysis of the existing lifestyle recommendations. This qualitative analysis provides insight into the level of evidence, strength of the recommendation, different elements of the lifestyle recommendations and knowledge gaps for future research. In our study, 11% of the recommendations were supported by a high or moderate level of evidence and 23% by (very) low evidence. These numbers are recognized by previous studies assessing the level of evidence from lifestyle recommendations, such as for the CPGs for dementia [32], polycystic ovary syndrome [33], paediatric depression [34] and the NICE guidelines [29]. The limited evidence support recommendations in CPGs is not restricted to lifestyle recommendations but extends to various types of recommendations, as assessed by the GRADE methodology. This trend was previously demonstrated in a cross‐sectional analysis of CPGs published by the National Clinical Effectiveness Committee (NCEC) [35]. This is why the GRADE working group is currently looking into additional methods to develop recommendations in the absence of high or moderate GRADE level of evidence [36, 37].

Our sensitivity analysis revealed a notable discrepancy between recommendation strength and the certainty of supporting evidence. While most recommendations were labelled as strong, they were often based on no or very low GRADE evidence. This discrepancy may be, in part, a reflection of the idea behind evidence‐based medicine (EBM), which values the best available evidence over certainty of supporting evidence alone, including experts' considerations and consensus [37]. In addition to recommendation strength, open coding revealed that strong recommendations are not always clearly actionable by GRADE standards. Effective guidance should consistently include three elements: actor(s), action(s) and indication(s). These components improve the clarity and practical use of CPGs, not just for lifestyle advice, but are generalizable to all CPG recommendations [38].

### 4.1 Future Directions

This study provided detailed insight into lifestyle recommendations in CPGs but did not examine the rationale behind guideline panel decisions. Given the lack of correlation between recommendation strength and evidence certainty (GRADE), the evidence‐to‐decision framework may offer further understanding. Additional research is needed to develop and validate tools for assessing the certainty of (suitable) evidence in the context of lifestyle [39]. We also identified key elements for making recommendations clear and actionable but did not evaluate their use in practice. It remains unclear under what conditions lifestyle recommendations are adopted, what barriers exist, and how implementation can be improved. Future studies will address these questions, incorporating input from both healthcare professionals and patients. In addition, this analysis highlighted several knowledge gaps in existing CPGs that can inform future research agendas. Many of these gaps concern general or combined lifestyle interventions for overweight and obesity. As identifying such gaps is not the primary goal of CPG development, additional gaps likely remain—for instance, those described in the strategic knowledge agenda of the Dutch Lifestyle in Healthcare Consortium [40].

### 4.2 Strengths and Limitations

A strength of this study was the systematic analysis method consisting of both quantitative and qualitative analyses. This led to an in‐depth analysis of not only the numbers of recommendations but also insights in the content, evidence and wording of these recommendations. In addition, we analysed a large number of CPGs concerning nine large disease groups (*n* = 130 CPGs), resulting in a large data set for in‐depth analysis. Another strength of this study concerns the global applicability of its methodology and findings. Although the analysis focused solely on Dutch CPGs, their use of the widely accepted and internationally adopted GRADE methodology ensures their generalizability. Finally, with the in‐depth analysis of the individual recommendations we identified clear outcomes and results for different stakeholders in guideline development. For policymakers, this analysis aids in prioritizing and substantiating lifestyle medicine. Because 62% of the recommendations were strongly formulated, there is high pressure for those recommendations to be implemented [41]. At the same time, future healthcare plans are being developed globally which supports momentum for change [42]. For medical specialists, this analysis helps to create awareness and implementation of existing lifestyle recommendations. For guideline developers, this creates awareness about the importance and need for improvements when it comes to including lifestyle recommendations in guidelines – for instance, following the GRADE methodology but also developing clear lifestyle recommendations which support implementation in clinical practice.

This in‐depth analysis also has limitations. First, findings of this cross‐sectional analysis are not based on all CPGs but are limited to the CPGs for medical specialists about the nine disease groups selected (cancer, cardiovascular diseases, respiratory diseases, depression, obesity, skin diseases, rheumatic diseases, diabetes and dementia). As lifestyle plays a significant role in the aetiology and/or treatment of this selection, numbers are expected to be higher when compared to assessing all CPGs. Second, we applied a pragmatic method to extract the recommendations from the selected CPGs by using the search bar in the online database with a limited sensitivity. Furthermore, the identification of lifestyle recommendations was divided over the two researchers (F.H., A.N.). Additionally, no intercoder reliability coefficient was calculated. To ensure the consistency and credibility of the qualitative coding, a structured process including jointly coding of a subset of the data, collaborative discussion about ambiguities or discrepancies and the involvement of a senior researcher.

### 4.3 Conclusion

This cross‐sectional analysis revealed the prevalence, evidence, content and wording of lifestyle recommendations in CPGs for chronic diseases in the medical specialist' care. First, we found that lifestyle recommendations were present in the majority (65%) of the CPGs reviewed, underscoring their recognized importance in chronic disease management. Second, 62% of these recommendations were strongly worded, but often lacked a clear clinical intent (actor(s), action(s) and indication(s)) to guide practice. Third, our findings reveal notable knowledge gaps regarding the effectiveness and feasibility of lifestyle interventions in daily practice. These findings emphasize that lifestyle recommendations are a fundamental part of chronic disease management, according to current CPGs. Similarly, they underscore the need for a better understanding on whether, why and how lifestyle recommendations are implemented into routine care.

## Author Contributions

**Florien Ham:** conceptualization, data curation, formal analysis, investigation, methodology, project administration, resources, software, validation, visualization, writing – original draft preparation. **Anh N. Nguyen:** conceptualization, data curation, formal analysis, investigation, methodology, project administration, resources, validation, writing – review and editing. **Elizabeth F. C. van Rossum:** supervision, writing – review and editing. **Mariëtte R. Boon:** supervision, writing – review and editing. **Janke F. de Groot:** conceptualization, funding acquisition, investigation, methodology, project administration, resources, supervision, validation, writing – review and editing.

## Conflicts of Interest

The authors declare no conflicts of interest.

## Supporting information


**Figure S1:** Illustration of the construction of CPGs. **Figure S2:** Formulas of the quantitative assessment of CPGs including lifestyle advice, lifestyle factors, the level of evidence and knowledge gaps. **Table S1:** List of lifestyle factors and synonyms used in the Dutch database for CPGs (including translation to English). **Table S2:** Overview and explanation of the different levels of evidence described in the Dutch CPGs.

## Data Availability

The data that support the findings of this study are available on request from the corresponding author. The data are not publicly available due to privacy or ethical restrictions.
