# Supplementary material for: Lifestyle Recommendations in the Dutch Clinical Practice Guidelines
Source: Clin Public Health Guidel. 2025 Sep 4;2(4):e70037. doi: 10.1002/gin2.70037 (PMC13563366; doi:10.1002/gin2.70037)
Supplement: Supplementary file 1 [file GIN2-2-e70037-s001.docx]

# **Supplementary material**

**Figure S1.** Illustration of the construction of CPGs.

CPGs including lifestyle advice = $\frac{CPGs with \geq1recommendation(s) about lifestyle (n)}{Selected sample of CPGs (n)}$ * 100%

Distribution of lifestyle factors mentioned $=\frac{Recommendations about lifestyle factor x (n)}{Total recommendations from selected sample of CPGs (n)}$ * 100%

The distribution of the level of evidence $=\frac{Recommendations with GRADE level of evidence X (n)}{Recommendations from selected sample of CPGs (n)}$ * 100%

Knowledge gaps about lifestyle: = $\frac{Knowledge gap in category X (n)}{Knowledge gaps in total (X)}$ * 100%

**Figure S2.** Formulas of the quantitative assessment of CPGs including lifestyle advice, lifestyle factors, the level of evidence and knowledge gaps.

| **Bewegen**  **(physical activity)** | **Roken**  **(smoking)** | **Alcohol/ middelen (alcohol/ substances)** | **Voeding**  **(nutrition)** | **Ontspanning**  **(relaxation)** | **Slaap**  **(sleep)** | **Leefstijl algemeen**  **(generic)** |
| --- | --- | --- | --- | --- | --- | --- |
| Fysieke activiteit (physical activity) | Rookgedrag (smoking behaviour) | Drinken (drinking) | Voedsel (nutrition) | Ontspannen (relax) | Slapen (sleeping) | Preventie (prevention) |
| Fysieke inspanning (physical effort) | Tabak (tabacco) | Dronken (drunk) | Dieet (diet) | Stress  (stress) | Nachtrust (night’s sleep) | Lifestyle (lifestyle) |
| Inspanning (exertion) | Rook  (smoke) | Middelen (substances) | Eet  (eat) | Rust  (rest) | Bedrust  (bed rest) | Welzijn  (well-being) |
| Beweging  (moving) |  | Drugs  (drugs) | Eetgedrag  (eating behaviour) | Zingeving (meaningfulness) | Slaappatroon (sleep pattern) | Leefstijl-verbetering  (lifestyle improvement) |
| Lichaamsbeweging  (exercise) |  |  | Inname  (intake) | Dagindeling (daily schedule) |  | Leefgewoonten (living habits) |
| Lichamelijke activiteit (physical activity) |  |  | Vitamine (vitamin) | Dagbesteding (daytime activities) |  | Leefregels (living rules) |
| Sport/sporten (sporting/sports) |  |  | Supplement (supplement) |  |  | Gewicht (weight) |
| Fysiotherapie (physiotherapy) |  |  | Eten  (food) |  |  | Afvallen (weight loss) |
| Ergotherapie (ergotherapy) |  |  | Voedingstherapie  (nutritional therapy) |  |  | BMI (body mass index) |
| Oefentherapie (exercise therapy) |  |  | Voedingssupplementen (nutritional supplements) |  |  | Gezonde leefstijl (healthy lifestyle) |
| Prehabilitatie (prehabilitation) |  |  | Voedingsinterventie (nutritional intervention) |  |  | Overgewicht (overweight) |
| Beweegprogramma  (exercise program) |  |  | Eetpatroon (eating pattern) |  |  | Obesitas (obesity) |
| Beweeginterventie  (exercise intervention) |  |  | Eetgewoonten (eating habits) |  |  | Psychosociale zorg (psychosocial care) |
|  |  |  | Voedingspatroon  (diet) |  |  |  |

**Table S1.** List of lifestyle factors and synonyms used in the Dutch database for CPGs (including translation to English).

**Table S2**. Overview and explanation of the different levels of evidence described in the Dutch CPGs.

| **GRADE** | **EBRO 1-4** | **EBRO A-D** | **Definition (according to GRADE)** |
| --- | --- | --- | --- |
| No | - | - | There is uncertainty that the true effect of treatment is close to the estimated effect of treatment. It is impossible to draw a conclusion, based on literature. |
| Very low | 4 | D | There is very low certainty that the true effect of treatment is close to the estimated effect of treatment. The literature conclusion is very uncertain. |
| Low | 3 | C | There is low certainty that the true effect of treatment is close to the estimated effect of treatment. There is a real chance that the conclusion will change in clinical relevance when results from new large-scale research are added to the literature analysis. |
| Moderate | 2 | B | There is reasonable certainty that the true effect of treatment is close to the estimated effect of treatment. It is possible that the conclusion will change in clinical relevance when results from new large-scale research are added to the literature analysis. |
| High | 1 | A | There is high certainty that the true effect of treatment is close to the estimated effect of treatment. It is very unlikely that the literature conclusion will change in clinical relevance when results from new large-scale research are added to the literature analysis. |
